# The Risk of Ventricular Arrhythmias between Alcohol Septal Ablation and Septal Myectomy in Hypertrophic Cardiomyopathy: A Meta-Analysis on Septal Reduction Therapy

**DOI:** 10.31083/j.rcm2312391

**Published:** 2022-11-30

**Authors:** Wei Tang, Menghui Liu, Jie Li, Rongxuan Chang, Chen Su, Xiaoyu Zhang, Lichun Wang

**Affiliations:** ^1^Department of Cardiology, The First Affiliated Hospital of Sun Yat-sen University, 510080 Guangzhou, Guangdong, China; ^2^Department of Medicine College, Sun Yat-sen University, 510080 Guangzhou, Guangdong, China

**Keywords:** hypertrophic cardiomyopathy, septal reduction therapy, alcohol septal ablation, septal myectomy, ventricular arrhythmias, meta-analysis

## Abstract

**Background:**

Alcohol septal ablation (ASA) has been more commonly applied in medical refractory hypertrophic obstructive cardiomyopathy (HOCM) compared with septal myectomy (SM), however its potential to create a proarrhythmic substrate is increased.

**Methods:**

A systematic search was performed in PubMed, EMBASE, Web of Science, and the Cochrane Library from inception to October 2020. Fixed or random effects models were used to estimate the risk ratios (RR) for ventricular arrhythmia events or other outcomes between the SM and ASA cohorts.

**Results:**

Twenty studies with 8025 patients were included. Pool analysis showed that the incidence of ventricular tachycardia (VT)/ventricular fibrillation (VF), which included appropriate implantable cardioverter defibrillator (ICD) intervention, was significantly higher in the ASA cohort than that in the SM cohort (ASA *vs* SM: 10% (345/3312) *vs* 5% (161/3227) (RR = 1.98, 95% CI (confidence interval), 1.65–2.37; *p *< 0.00001, $I^{2}$ = 0%). In both groups, more than 90% of VT/VF events occurred in the early phase (during the procedure, during hospitalization or within 30 days after the procedure) (ASA: 94.20%; SM: 94.41%). Further subgroup analysis also showed that the ASA group had a higher incidence of VT/VF in both the early phase (RR = 1.94, 95% CI, 1.61–2.33; *p *< 0.0001, $I^{2}$ = 0%) and the late phase (RR = 2.80, 95% CI, 1.00–7.89; *p* = 0.05, $I^{2}$ = 33%). Furthermore, although the risks of sudden cardiac death (SCD) were similar between the ASA and SM groups, a higher incidence of sudden cardiac arrest (SCA), which included SCD and resuscitated SCA, was observed in the ASA group (RR = 2.30, 95% CI, 1.35–3.94; *p* = 0.002, $I^{2}$ = 0%).

**Conclusions:**

In patients with HOCM, those who received ASA showed a higher incidence of VF/VT and SCD combined with resuscitated SCA. The majority of VT/VF occurred in the early phase.

## 1. Introduction

Hypertrophic cardiomyopathy (HOCM) is the most common inherited cardiovascular disease. The majority of patients have an abnormally thickened ventricular septum, which could lead to systolic anterior motion of the mitral valve and obstruction of the left ventricular outflow tract (LVOT) [1]. Initial pharmacological therapy including beta-blockers and verapamil produces a negative inotropic effect to relieve the obstruction [2, 3]. In patients who are refractory to medical treatments, septal reduction therapy (SRT) is indicated. Surgical septal myectomy (SM) and alcohol septal ablation (ASA) are the two most common SRTs [4]. Although there is no randomized controlled study (RCT) comparing these two treatments, an increasing number of ASAs are being performed due to its reduced invasiveness [5].

Unlike the direct resection of the hypertrophic cardiac muscle, ASA works by inducing an iatrogenic myocardial infarction. In theory, it could be a potential substrate for ventricular arrhythmias and sudden cardiac deaths (SCD) [6, 7]. Although the possible proarrhythmic properties of ASA has been proposed since its emergence [8], this has not been substantiated in more recent studies [9, 10]. Furthermore, previous meta-analyses or systematic reviews comparing SM and ASA, such as the latest two from Mohammed *et al*. [11] and Ibadete *et al*. [12] in 2019 and 2020, respectively, rarely compared ventricular arrhythmic events. Therefore, in the current study, we focused on the incidence of post-procedure ventricular arrhythmic events between these two therapies. The aim was to analyze whether ASA would increase the risk of ventricular arrythmias.

## 2. Methods

The preferred reporting items for systematic reviews and meta-analysis (PRISMA) statement was followed in this meta-analysis. Due to the study design, neither institutional review board (IRB) approval nor informed patient consent was needed.

### 2.1 Literature Search

A systematic search was performed in PubMed-Medline, EMBASE, the Cochrane Library and Web of Science databases using the terms “hypertrophic obstructive cardiomyopathy”, “idiopathic hypertrophic sub-aortic stenosis”, “asymmetric septal hypertrophy”, “septal reduction therapy”, “septal myectomy”, “Morrow septal myectomy”, “modified morrow septal myectomy”, “alcohol septal ablation” and “percutaneous transluminal myocardial ablation”. No time limit to the start date was applied, and the search was conducted up to October 2020. The detailed search strategies are presented in **Supplementary Table 1**. The inclusion criteria included (a) studies comparing the outcomes of ASA and SM; (b) enrolled patients ≥18 years; (c) published language restricted to English or a complete English translation version; and (d) follow-up the studies were more than 30 days.

### 2.2 Data Extraction

Two examiners (WT, ML) independently screened the titles and abstracts (if available) of the entries identified in different databases. Next, the full text of all studies that met the eligibility criteria or those with insufficient information from the titles or abstracts to make a decision, were obtained for the next screening phase. All studies that did not meet the criteria were excluded, and the reasons for exclusion were noted. Case reports and series, review articles, editorials and duplicate reports were excluded. For those studies that reported the same study or utilized the repeated data at different follow-up intervals, we pooled all the relevant details together and used the most comprehensive data for further analysis. A third member (JL) would further check the data whenever there was disagreement until a consensus was reached. Data from the included studies were then extracted by two independent reviewers (WT, ML) based on a predesigned outline. The extracted information included the design of the study, study population (number of participants, age, and sex), length of follow-up, clinical characteristics and outcomes such as the pre- and post-procedure left ventricular outflow tract pressure gradient, sustained ventricular tachycardia or ventricular fibrillation (VT/VF) events (including appropriate implantable cardioverter defibrillator (ICD) intervention) during and post-procedure, SCD and resuscitated sudden cardiac arrest (SCA) events post-procedure, the ICD implantation rate and permanent pacemaker (PPM) implantation rate post procedure, the reintervention rate and all-cause or cardiac mortality post procedure. Additional details are presented in Table 1 (Ref. [10, 13, 14, 15, 16, 17, 18, 19, 20, 21, 22, 23, 24, 25, 26, 27, 28, 29, 30, 31]). VF/VT events were divided into an early-phase (which occurred during the procedure, during hospitalization or within 30 days after the procedure) and a late-phase (which occurred >30 days after the procedure and out of hospital) subgroup.

**Table 1.** **Clinical characteristics of enrolled ${\mathrm{studies}}^{a}$**.

Study, year	No. of patients (n)		Age (mean ± SD)/mean (range)		Male (%)		Resting LVOT PG (mmHg)		Post-procedure LVOT PG (mmHg)		Prior ICD (n)		Follow-up (years)	Reported outcomes
	ASA	SM	ASA	SM	ASA	SM	ASA	SM	ASA	SM	ASA	SM		
Nagueh SF, 2001 [31]	41	41	49 ± 17	49 ± 16	NA	NA	76 ± 23	78 ± 30	8 ± 15	4 ± 7	NA	NA	1	Mortality, PPM/ICD, VA
Qin JX, 2001 [30]	25	26	63 ± 14	48 ± 13	28	62	64 ± 39	62 ± 43	28 ± 29	7 ± 7	NA	NA	0.25	Mortality, NYHA class, Reintervention, PPM
Firoozi S, 2002 [29]	20	24	49 ± 13	38 ± 16	60	54	91 ± 18	83 ± 23	22 ± 14	15 ± 10	NA	NA	2	Mortality, PPM, NYHA class
Jiang TY, 2004 [28]	43	11	45 (13–74)	36 (11–69)	NA	NA	76 ± 33	95 ± 48	20 ± 18	12 ± 18	NA	NA	2	Mortality, NYHA class, VA
Ralph-Edwards A, 2005 [27]	54	48	59 ± 15	46 ± 17	48	62	74 ± 36	64 ± 27	15 (0, 96)	5 (0, 17)	NA	NA	2.2	Mortality, PPM
Van der Lee C, 2005 [26]	43	29	52 ± 17	44 ± 12	NA	NA	101 ± 34	100 ± 20	23 ± 19	17 ± 14	NA	NA	1	Mortality, NYHA class, PPM, VA, reintervention
Valeti US, 2007 [25]	24	24	62 ± 12	50 ± 20	50	62.5	76 ± 40	75 ± 41	7 ± 6	3 ± 3	NA	NA	1.2	CMR outcomes, VA, PPM
Ten Cate FJ, 2010 [24]	91	40	54 ± 15	49 ± 15	55	53	92 ± 25	86 ± 19	NA	NA	0	4	5.4	Mortality, SCD, VA, PPM
Sorajja P, 2012 [23]	177	177	63 ± 13	62 ± 12	32	32	70 ± 40	67 ± 40	NA	NA	8	12	5	Mortality, SCD, VA, PPM
Steggerda RC, 2014 [21]	161	102	59 ± 14	56 ± 16	53	46	32 (18–75)	50 (25–75)	10 (7–19)	9 (4–10)	4	3	5.1	Mortality, NYHA class, VA, reintervention
Samardhi H, 2014 [22]	47	23	57 ± 14.7	47 ± 20.6	55	43.5	74.0 ± 59.5	75.5 ± 38.4	27.2 ± 37.5	12.9 ± 27.0	3	3	2	Mortality, NYHA class, VA, reintervention, PPM
Vriesendorp PA, 2014 [20]	321	253	58 ± 14	52 ± 16	55	54	102 ± 52	92 ± 39	10 ± 24	9 ± 16	NA	NA	7.5	Mortality, SCD, VA
Sedehi D, 2015 [19]	52	171	57.3 ± 12.9	48.0 ± 17.1	56	49	67.1 ± 26.9	67.4 ± 43.4	23.9 ± 29.4	11.2 ± 16.4	6	0	3.2	NYHA class, survival, PPM
Yang YJ, 2016 [18]	22	37	45.5 ± 8.1	44.6 ± 95	80	67	79.7 ± 21.2	69.0 ± 23.9	43.7 ± 28.9	15.0 ± 16.9	0	0	1	NYHA class, CMR outcomes, VA
Cavigli L, 2018 [17]	55	71	49 ± 14	42 ± 16	42	62	70 ± 33	52 ± 31	22 ± 21	11 ± 10	1	6	5	Mortality, SCD, ICD/PPM, reintervention
Guo HC, 2018 [16]	68	158	42 ± 16	37 ± 151	63	49	70.30 ± 44.79	74.58 ± 45.52	39.78 ± 22.07	13.95 ± 9.94	NA	NA	2	Mortality, VA, ICD/PPM, reintervention
Nguyen A, 2019 [10]	167	334	65 ± 14	64 ± 13	44.3	45.8	65 (29–100)	60 (32–85)	5 (0–15)	0.0 (0.0–3.0)	NA	NA	2.6	NYHA class, Survival, ICD/PPM
Kimmelstiel C, 2019 [15]	99	378	66.3 ± 11.9	52.7 ± 14.7	37	58	65.7 ± 40.7	58.0 ± 41.8	NA	NA	NA	NA	4.0	Mortality, NYHA class, ICD/PPM, VA, reintervention
Lemor A, 2020 [13]	2245	2113	62.0 ± 13.6	53.5 ± 13.6	41.3	46.5	NA	NA	NA	NA	245	285	5	Mortality, VA, ICD/PPM
Afanasyev AV, 2020 [14]	105	105	52.2 ± 14	51.9 ± 14.3	52.4	54.3	72 (48–90)	78 (63–90)	10 (0–20)	12 (8–20)	NA	NA	4	Mortality, SCD, PPM, Reintervention

^a^ Values are presented as means ± SD or medians (25–75 percentiles) for non-normally distributed data. Abbreviations: LVOT, left ventricular outflow tract; PG, pressure gradient; ICD, implantable cardioverter defibrillator; ASA, alcohol septal ablation; SM, septal myectomy; NYHA, New York heart association; VA, ventricular arrhythmias; SCD, sudden cardiac death; PPM, permanent peacemaker; CMR, cardiac magnetic resonance.

### 2.3 Quality Assessment

Since all of the included studies were observational, the assessment of the risk of bias was evaluated by a modified version of the Newcastle-Ottawa scale (NOS), which is a quality assessment tool for nonrandomized studies in three domains: the selection of participants, comparability of study groups, and the outcome of interest. The risk of bias in each study was evaluated by calculating the aggregate score on the 9 items. The detailed assessment of each study is shown in **Supplementary Table 2**.

### 2.4 Statistical Analysis

The data analysis was conducted by Review Manager 5.4 (The Cochrane Collaboration, Oxford, England) and Stata (version 15.1, StataCorp, College Station, TX, USA). Continuous variables were reported as the means ± standard deviation (SD) if they were normally distributed; otherwise, they were reported as medians and interquartile ranges (IQRs). The pooled effects are presented as the relative ratio (RR) or weighted mean difference (WMD) with a 95% confidence interval (CI), and sensitivity analyses were performed when significant heterogeneity was observed. The heterogeneity between studies was considered significant with an $I^{2}$ of more than 50% and a *p* value less than 0.05. The meta-analyses were performed with the fixed model when the heterogeneity between studies was not significant; otherwise, the randomized effect model was used, and sensitivity analyses were also needed. Publication bias was evaluated using funnel plots and Egger’s test. *p <* 0.05 was considered statistically significant.

## 3. Results

### 3.1 Search Results

**Fig. 1.**
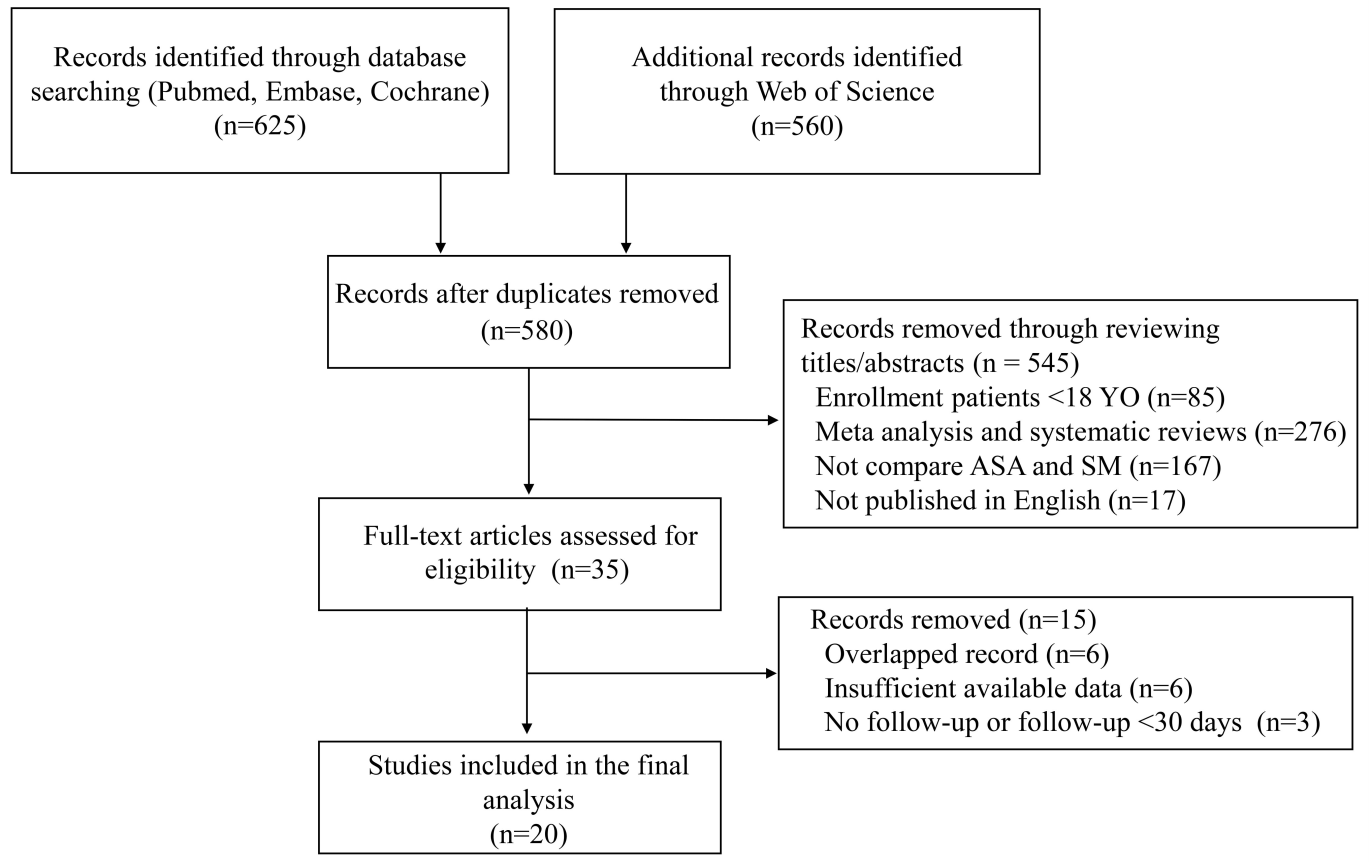
**Flow chart of literature search**.

A total of 1185 articles were initially retrieved from PubMed, Embase, the Web of Science, and the Cochrane Library. After removing duplicates, 580 articles were left for title and abstract review. Thirty-five full-text articles were further assessed for eligibility, and 20 studies were ultimately included for data extraction and analysis [10, 13, 14, 15, 16, 17, 18, 19, 20, 21, 22, 23, 24, 25, 26, 27, 28, 29, 30, 31] (Fig. 1). All studies were observational studies. The risk of bias was evaluated by the Newcastle Ottawa Scale (**Supplementary Table 2**). Among them, 5 studies acquired 6/9 points, 12 studies acquired 7/9 points, and the remaining 3 studies acquired 8/9 points.

### 3.2 Study Characteristics

The study characteristics are presented in Table 1. A total of 8025 patients were included in the 20 studies. Among them, 3860 patients received ASA treatment, and the remaining 4165 patients underwent SM. The mean follow-up periods varied from 3 months to 120 months. Of the 20 enrolled studies, twelve studies had a specific description of VT/VF events (including appropriate ICD intervention), eight studies contained descriptions about SCD and resuscitated SCA, and ten studies collected data about ICD implantation. The baseline LVOT pressure gradient was higher in the ASA groups (WMD = 5.89; 95% CI: 3.03–8.75; *p *< 0.0001; $I^{2}$ = 1%, **Supplementary Fig. 1a**). The baseline interventricular septal diameter (IVSd) in diastole was slightly thinner in the ASA groups (WMD = –0.68; 95% CI: –1.35, –0.02; *p* = 0.04; $I^{2}$ = 43%, **Supplementary Fig. 2a**), while the number of patients in NYHA class III/IV and the left ventricular end-diastolic diameter (LVEDd) prior to the intervention were similar between the two groups (*p* = 0.36, *p* = 0.51, **Supplementary Figs. 1b,2b**).

### 3.3 Analysis of VF/VT Events

**Fig. 2.**
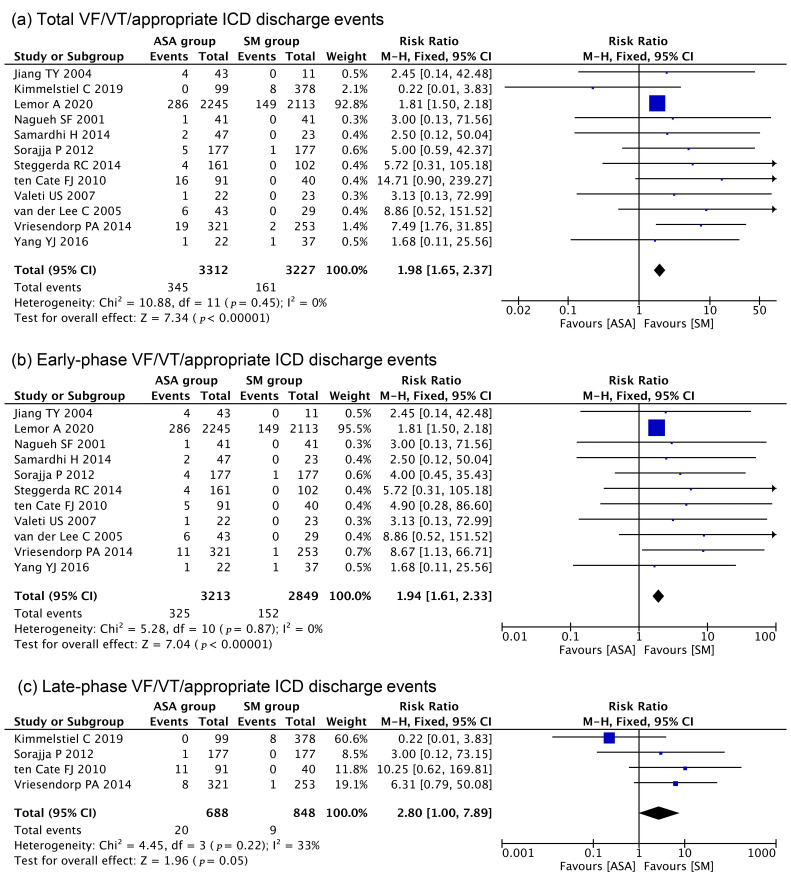
**Comparisons of ventricular fibrillation (VT)/ventricular tachycardia (VF)/appropriate ICD intervention events between ASA groups and SM groups**. (a) Total VF/VT events. (b) Early-phase VF/VT events. (c) Late-phase VF/VT events.

Among the 20 enrolled studies, 12 studies contained descriptions of VT/VF events (including ICD intervention). The pooled analysis showed that the incidence of total VT/VF events was almost twice as high in the ASA group (345/3312, 10.42%) than in the SM group (61/3227 patients, 4.99%) (RR = 1.98; 95% CI: 1.65–2.37; *p <* 0.0001; $I^{2}$ = 0%, Fig. 2a). When VT/VF events were classified as early-phase and late-phase, the data showed that more than 90% of VT/VF events occurred in the early-phase in both groups (ASA: 94.20%; SM: 94.41%). Further subgroup analysis indicated that VT/VF was significantly higher in the ASA group than in the SM group in both the early phase (RR = 1.94; 95% CI: 1.61–2.33; *p *< 0.0001; $I^{2}$ = 0%, Fig. 2b) and the late phase (RR = 2.80; 95% CI: 1.00–7.89; *p* = 0.05; $I^{2}$ = 33%, Fig. 2c). The sensitivity analysis of the enrolled studies demonstrated that the removal of each of them did not change the result of the pooled analysis (**Supplementary Table 3**). In addition, possible publication bias was not found in the funnel plot (**Supplementary Fig. 3**) or Egger’s test (*p* = 0.101). Further meta regression showed no significant interaction between the incidence of VT/VF with LVOT pressure gradient reduction (*p* = 0.904/0.220), with baseline ejection fraction (EF) (*p* = 0.552/0.685), with baseline IVSd (*p* = 0.799/0.054), and with baseline NYHA class III/IV proportion (*p* = 0.165/0.364) in both ASA and SM cohorts (**Supplementary Figs. 5–8**).

### 3.4 Analysis of SCD and SCA

**Fig. 3.**
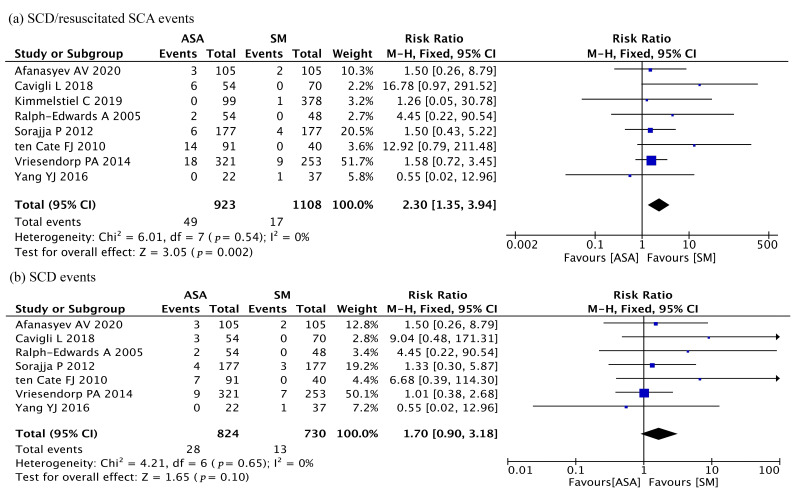
**Comparison of the incidence of sudden cardiac death (SCD)/resuscitated sudden cardiac arrest (SCA) after ASA and SM**. (a) Combined SCD/resuscitated SCA events. (b) SCD events alone.

The SCD and resuscitated SCA were combined as SCA to estimate the risk for SCD together in this analysis. In the 8 studies that described SCD and/or resuscitated SCA events, a total of 49 events were reported in the ASA group (SCD: 26; resuscitated SCA: 23), and 17 events were reported in the SM group (SCD: 13; resuscitated SCA: 4). The pooled analysis showed that the ASA group had a higher incidence of SCA (RR = 2.30; 95% CI: 1.35–3.94;* p* = 0.002; $I^{2}$ = 0%, Fig. 3a). However, when SCD was considered alone, the pooled analysis showed no significant difference between the two groups (ASA cohorts: 28/824, 3.40%; SM cohorts: 13/730, 1.8%; RR = 1.70; 95% CI: 0.90–3.18;* p* = 0.10; $I^{2}$ = 0%, Fig. 3b). Possible publication bias was also not found in the funnel plot (**Supplementary Fig. 4**) or Egger’s test (*p* = 0.667).

### 3.5 Analysis of All-Cause Mortality

All-cause mortality within 30 days and above 30 days after receiving ASA or SM was regarded as early-phase and late-phase mortality, respectively. For the ASA cohorts, there were 38 early-phase deaths and 89 late-phase deaths among 3361 (1.13%) and 1055 patients (8.43%), respectively, while in the SM cohorts, there were 75 early-phase deaths and 88 late-phase deaths among 3520 (2.13%) and 1304 patients (6.75%), respectively. The pooled analysis showed no significant differences between ASA and SM in either early-phase mortality (RR = 0.72, 95% CI: 0.37–1.41; *p* = 0.34; $I^{2}$ = 31%) or late-phase all-cause mortality (RR = 1.04, 95% CI: 0.63–1.69; *p *= 0.89; $I^{2}$ = 39%) (Table 2).

**Table 2.** **The difference of risks of various post-procedure outcomes in patients received ASA or SM**.

Post-procedure outcomes	Number of studies	ASA group		SM group		RR	95% CI	*p* value	$I^{2}$, %
		Event	Total patients	Event	Total patients				
Early-phase death	11	38	3361	75	3520	0.72	0.37–1.41	0.34	31.00
Late-phase death	9	89	1055	88	1304	1.04	0.63–1.69	0.89	39.00
PPM implantation	17	404	3474	240	3864	1.99	1.39–2.83	<0.01	50.00
ICD implantation	10	123	1208	89	1589	1.67	0.98–2.86	0.06	64.00
Reintervention	8	68	603	5	892	10.50	5.10–21.64	<0.01	0.00

Abbreviation: ASA, alcohol septal ablation; SM, septal myectomy; PPM, permanent peacemaker; ICD, implantable cardioverter defibrillator; RR, risk ratio; CI, confidence interval.

### 3.6 Analysis of Pacemaker and ICD Implantation and Reintervention after the Procedure

After ASA, 404 patients were implanted with a permanent pacemaker in the 3474 pooled patients (11.63%), which was significantly higher compared with (240/3864, 6.21%) after SM (RR = 1.99; 95% CI: 1.39–2.83; *p* = 0.0002; $I^{2}$ = 50%; Table 2). In addition, the pooled analysis indicated that patients receiving ICD implantation were not significantly different between the two cohorts (ASA group: 123/1208, 10.18%; SM group: 89/1589, 5.60%; RR = 1.67; 95% CI: 0.98–2.86;* p* = 0.06; $I^{2}$ = 64%, Table 2). The reintervention rate was significantly higher in the ASA group than in the SM group (ASA *vs* SM: 11.28% *vs* 0.56%; RR = 10.50; 95% CI: 5.10–21.64; *p *< 0.0001; $I^{2}$ = 0%; Table 2).

## 4. Discussion

Our results showed that the risk of ventricular arrhythmias (VT/VF and appropriate ICD intervention) and SCA (SCD and resuscitated SCA) were higher in the ASA cohort than in the SM cohort. Additionally, pacemaker implantation and reintervention were required more often in the ASA group, but there was no significant difference in all-cause mortality between the two groups.

Due to several metabolic, autonomic, and electrophysiological changes, myocardial infarction can be arrhythmogenic and is closely related to lethal arrhythmias and SCD [32]. Hence the iatrogenic myocardial infarction resulting from ASA increases the potential risk for life-threatening arrhythmias [1, 6, 7, 8]. However, in recent years, these concerns have not been substantiated [9, 33]. Therefore, the latest 2020 American Heart Association/American College of Cardiology guideline still indicate that further studies are needed [34]. In this analysis, we compared the incidence of VT/VF between the ASA and SM groups. The results showed that the ASA cohort had a higher risk of VT/VF, even when it was divided into the early phase (during the procedure, during hospitalization or within 30 days after the procedure) and late phase (>30 days after the procedure and out of the hospital). In addition, we found that more than 90% of VT/VF events occurred in the early phase. This indicates that the proarrhythmic risk should not be neglected after ASA, especially in the early-phase after the procedure. Other studies have also reported this phenomenon. Balt JC *et al*. [35] found that sustained VT or VF attacks were recorded only within 30 days after ASA by continuous rhythm monitoring. The generation of heterogeneous iatrogenic intramyocardial scars is generally believed to be the mechanism for VT/VF after ASA [35]. A previous study reported that the VT attacks in HOCM patients who did not receive invasive therapies were predominantly polymorphic [36], while the VT recorded after ASA often manifested as monomorphic VT [36, 37]. This finding indicates that VT/VF after ASA is probably a pattern of re-entrant arrhythmias related to the iatrogenic intramyocardial scar. In addition, the high reintervention rate in ASA may indicate that the reduction in the LVOT pressure gradient was not ideal in some patients. This might explain the higher incidence of VT/VF in the ASA cohort; since a previous study suggested that the relief of the LVOT pressure gradient would decrease the appropriate ICD discharge by improving cardiac haemodynamics [38].

SCD is one of the most devastating complications of HOCM. Most recent studies and meta-analyses showed no significant difference in SCD between ASA and SM [39]. In this analysis, we also did not find that the incidence of SCD was significantly different in the ASA and SM groups. However, when we regarded SCD and resuscitated sudden cardiac arrest (SCA) as a single event (SCA), the ASA group had more than twice the incidence than the SM group. This suggests that patients receiving ASA were more likely to be exposed to SCD and more dependent on effective resuscitation or ICD intervention. This was consistent with the higher incidence of malignant ventricular arrhythmias after ASA.

Our meta-analysis also found some results consistent with those of previous studies [11, 39]. ASA is as effective as SM in decreasing LVOT pressure and relieving obstructive symptoms. Patients receiving ASA are more likely to require permanent peacemakers and reinterventions. ASA and SM carry similar low risk for early- or late-phase mortality. Both therapies tend to be more effective in reducing symptoms than medical management alone. In those patients who are refractory to adequate medical therapy, the results of our meta-analysis may be helpful in determining the selection of ASA *vs* SM for an individual patient in order to achieve the best clinical outcomes.

## 5. Limitations

There are several limitations in this study. First, the absence of an RCT to compare ASA and SM inevitably brings about the concern for selection bias since we conducted a sensitivity analysis regarding the incidence of VF/VT, which further supports our results. In addition, as the study from Lemor *et al*. [13] collected the data from the National Readmission Database, its study size was much larger than that of other studies. Thus, it was weighted as the absolute majority in the pooled analysis, which could lead to some bias; however, this concern could also be relieved by the sensitivity analysis, where we could obtain the same result even if this study were excluded from the cohorts. Another limitation is that we included some studies from 20 years ago. Since that time, there have been significant improvements in both but the development of ASA and SM techniques; however, Liebregts *et al*. [39] found no association between the study period and all-cause mortality.

## 6. Conclusions

Patients with HOCM who underwent ASA had nearly more than twice the risk of VF/VT events. Most events occurred during the procedure or during hospitalization or within 30 days post-procedure. In addition, patients who receive ASA were more likely to be exposed to SCD and resuscitated SCA.

## Abbreviations

ASA, Alcohol septal ablation; SM, Septal myectomy; SRT, Septal reduction therapy; HOCM, Hypertrophic obstructive cardiomyopathy; LVOT, Left ventricular outflow tract; VT, Ventricular tachycardia; VF, Ventricular fibrillation; SCD, Sudden cardiac death; SCA, Sudden cardiac arrest; ICD, Implantable cardioverter defibrillator; PPM, Permanent pacemaker; IVSd, Intraventricular septal diameter; RR, Risk ratio; WMD, Weighted mean difference; CI, Confidence interval; NYHA, New York Heart Association.

## Supplementary Material

Supplementary material associated with this article can be found, in the online version, at https://doi.org/10.31083/j.rcm2312391.
