# Supplementary material for: The Risk of Ventricular Arrhythmias between Alcohol Septal Ablation and Septal Myectomy in Hypertrophic Cardiomyopathy: A Meta-Analysis on Septal Reduction Therapy
Source: Rev Cardiovasc Med. 2022 Nov 30;23(12):391. doi: 10.31083/j.rcm2312391 (PMC11270481; doi:10.31083/j.rcm2312391)

Supplemental Material

Table S1. Searching strategies in one of the databases.

Table S2. Results of quality assessment of included studies using Newcastle-OttawaQuality Assessment Scale (NOS).

Table S3. The sensitive analysis of ventricular fibrillation (VF)/ventricular tachycardia(VT)/ appropriate ICD discharge events.

Figure S1. The comparisons of pre procedure left ventricular outflow tract (LVOT) pressure gradient (mmHg) and NYHA class III/IV patients between ASA groups and SM groups; (a) pre-procedure LVOT pressure gradient; (b) NYHA class III/IV.

Figure S2. The comparisons of baseline interventricular septal diameter (IVSd, mm) and left ventricular end diastolic diameter (LVEDd, mm) between ASA groups and SM groups; (a) IVSd; (b) LVEDd.

Figure S3. valuation of publication bias in the studies comparing ventricular arrythmiasby funnel plot. RR, risk ratio.

Figure S4. Evaluation of publication bias in the studies comparing sudden cardiac death (SCD)/resuscitated sudden cardiac arrest (SCA) by funnel plot. RR, risk ratio.

Figure S5. Meta-regression for interaction of LVOT pressure gradient reduction with VT/VF incidence between ASA and SM groups.

Figure S6. Meta-regression for interaction of baseline EF (%) with VT/VF incidence between ASA and SM groups.

Figure S7. Meta-regression for interaction of baseline IVSd (mm) with VT/VF incidence between ASA and SM groups.

Figure S8. Meta-regression for interaction of baseline NYHA class III/IV proportion (%) with VT/VF incidence between ASA and SM groups.

Table S1. Searching strategies in one of the databases.

Searching order	Typed text
#1	Hypertrophic obstructive cardiomypathy
#2	Idiopathic hypertrophic sub-aortic stenosis
#3	Asymmetric septal hypertrophy
#4	#3 OR #2 OR #1
#5	Septal reduction therapy
#6	Septal myectomy
#7	Morrow septal myectomy
#8	Modified morrow septal myectomy
#9	#8 OR #7 OR #6 OR #5
#10	alcohol septal ablation
#11	Percutaneous transluminal myocardial ablation
#12	#11 OR #12
#13	#12 AND #9 AND #4

Table S2. Results of quality assessment of included studies using Newcastle-Ottawa Quality Assessment Scale (NOS).

Study/year	1	2	3	4	5A	5B6		7 8		Score
Nagueh SF, 2001	★	★	★	_	★	★	★	★		7
Qin JX, 2001	★	★	★	_	_	_	★	★	★	6
Firoozi S, 2002	★	★	★	_	★	★	★	★_		7
Jiang TY, 2004	★	★	★	_	★	_	★	★_		6
Ralph-Edwards A, 2005	★	★	★	_	★	★	★	_	★	7
Van der Lee C, 2005	★	★	★	_	★	★	★	★_		7
Valeti US, 2007	★	★	★	_	★	★	★	★_		7
Ten Cate FJ, 2010	★	★	★	_	★	_	★	★	★	7
Sorajja P, 2012	★	★	★	_	★	★	★	★	★	8
Steggerda RC, 2014	★	★	★	_	★	_	★	★_		6
Vriesendorp PA, 2014	★	★	★	_	★	_	★	★	★	7
Samardhi H, 2014	★	★	★	_	★	_	★	★_		6
Sedehi D, 2015	★	★	★	_	★	★	★	★_		6
Yang YJ, 2016	★	★	★	_	★	_	★	★	★	7
Cavigli L, 2018	★	★	★	_	★	_	★	★	★	7
Guo HC, 2018	★	★	★	_	★	_	★	★	★	7
Nguyen A, 2019	★	★	★	_	★	★	★	★_		7
Kimmelstiel C, 2019	★	★	★	_	★	★	★	★	★	8
Lemor A, 2020	★	★	★	_	★	★	★	★_		7
Afanasyev AV, 2020				_	★	★	★	★	★	8

(1) Indicates the exposed cohort was truly representative; (2) the non-exposed cohort was drawn from the same community; (3) ascertainment of the exposure; (4) the outcome of interest was not present at the start of the study; (5A) cohorts were comparable on the basis of diagnosis of obstructive hypertrophic cardiomyopathy. (5B) cohorts were comparable under the matching of other baseline characteristics. (6) quality of outcome assessment; (7) Follow-up was long enough for outcome to occur; (8) complete follow-up (> 80% follow-up or description of those lost).

Table S3. The sensitive analysis of ventricular fibrillation/ventricular tachycardia/appropriate ICD discharge events.

Study	Risk ratio	Confidence interval
Nagueh SF, 2001	1.97	1.64-2.37
Jiang TY, 2004	1.97	1.64-2.37
van der Lee C, 2005	1.95	1.63-2.34
Valeti U, 2007	1.97	1.64-2.37
ten Cate FJ, 2010	1.92	1.60-2.31
Sorajja P, 2012	1.96	1.63-2.35
Samardhi H, 2014	1.97	1.64-2.37
Vriesendorp PA, 2014	1.90	1.58-2.28
Steggerda RC, 2014	1.96	1.63-2.35
Yang YJ, 2016	1.98	1.65-2.37
Kimmelstiel C, 2019	2.01	1.68-2.42
Lemor A, 2020	4.16	2.12-8.16

Figure S1. The comparison of pre procedure left ventricular outflow tract (LVOT) pressure gradient (mmHg) between ASA groups and SM groups

(a) comparison of pre-procedure LVOT pressure gradient.

	ASA group			SM group				Mean Difference
Study or Subgroup	Mean	SD	Total	Mean	SD	Total	Weight	IV, Fixed, 95% CI
Cavigli L 2018	70	33	55	52	31	71	6.4%	18.00 [6.68, 29.32]
Firoozi S 2002	91	18	20	83	23	24	5.6%	8.00 [−4.12, 20.12]
Guo HC 2018	70.3	44.79	68	74.58	158	158	1.1%	−4.28 [−31.12, 22.56]
Jiang TY 2004	76	33	43	95	48	11	0.9%	−19.00 [−49.03, 11.03]
Kimmelstiel C 2019	65.7	40.7	99	58	41.8	378	10.0%	7.70 [−1.36, 16.76]
Nagueh SF 2001	76	23	41	78	30	41	6.1%	−2.00 [−13.57, 9.57]
Qin JX 2001	64	39	25	62	43	26	1.6%	2.00 [−20.51, 24.51]
Ralph-Edwards A 2005	74	36	54	64	27	48	5.4%	10.00 [−2.27, 22.27]
Samardhi H 2014	74	59.5	47	75.5	38.4	23	1.5%	−1.50 [−24.64, 21.64]
Sedehi D 2015	67.1	26.9	52	67.4	43.4	171	8.6%	−0.30 [−10.09, 9.49]
Sorajja P 2012	70	40	177	67	40	177	11.8%	3.00 [−5.33, 11.33]
ten Cate FJ 2010	92	25	91	86	19	40	13.4%	6.00 [−1.81, 13.81]
Valeti US 2007	76	40	24	75	41	24	1.6%	1.00 [−21.92, 23.92]
van der Lee C 2005	101	34	43	100	20	29	5.2%	1.00 [−11.50, 13.50]
Vriesendorp PA 2014	102	52	321	92	39	253	14.8%	10.00 [2.55, 17.45]
Yang YJ 2016	79.7	21.2	22	69	23.9	37	5.9%	10.70 [−1.04, 22.44]
**Total (95% CI)**			**1182**			**1511**	**100.0%**	**5.89 [3.03, 8.75]**

Heterogeneity: $Chi^2 = 15.16$, df = 15 ($p = 0.44$); $I^2 = 1\%$
Test for overall effect: Z = 4.03 ($p < 0.0001$)

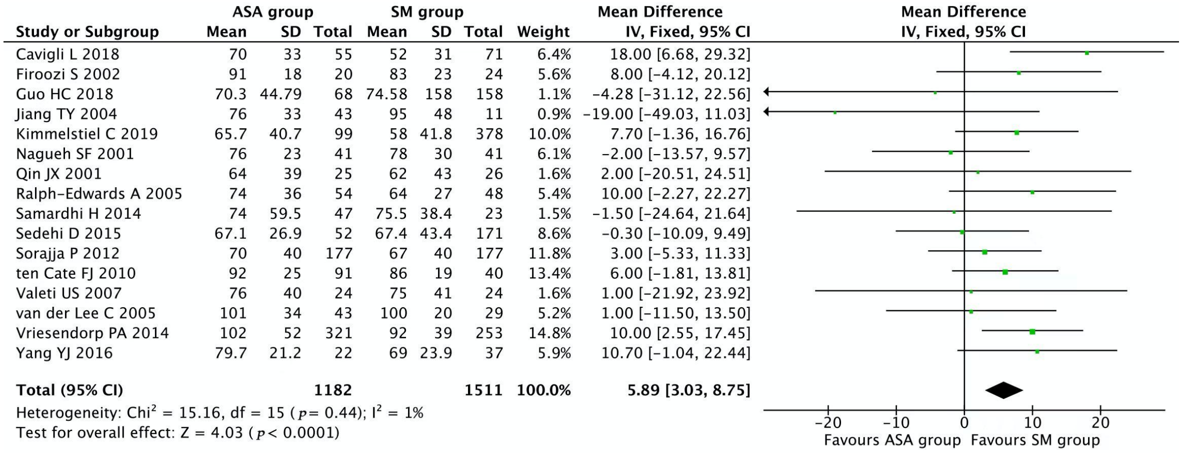

(b) comparison of baseline NYHA III/IV patients.

	ASA group		SM group			Odds Ratio
Study or Subgroup	Events	Total	Events	Total	Weight	M-H, Random, 95% CI
Afanasyev AV 2020	71	150	222	345	13.5%	0.50 [0.34, 0.73]
Firoozi S 2002	6	20	9	24	9.5%	0.71 [0.20, 2.53]
Guo HC 2018	8	68	5	158	10.0%	4.08 [1.28, 12.97]
Kimmelstiel C 2019	94	99	352	378	10.9%	1.39 [0.52, 3.71]
Nagueh SF 2001	41	41	32	41	4.0%	24.26 [1.36, 432.48]
Samardhi H 2014	23	47	12	23	10.8%	0.88 [0.32, 2.38]
Sorajja P 2012	177	177	133	177	4.2%	118.33 [7.22, 1938.89]
Steggerda RC 2014	80	161	76	102	13.0%	0.34 [0.20, 0.58]
Vriesendorp PA 2014	249	321	165	253	13.5%	1.84 [1.28, 2.67]
Yang YJ 2016	13	22	21	37	10.5%	1.10 [0.38, 3.21]
**Total (95% CI)**		**1106**		**1538**	**100.0%**	**1.37 [0.69, 2.72]**
Total events	762		1027			

Heterogeneity: $Tau^2 = 0.86$; $Chi^2 = 64.48$, df = 9 ($p < 0.00001$); $I^2 = 86\%$
Test for overall effect: Z = 0.91 ($p = 0.36$)

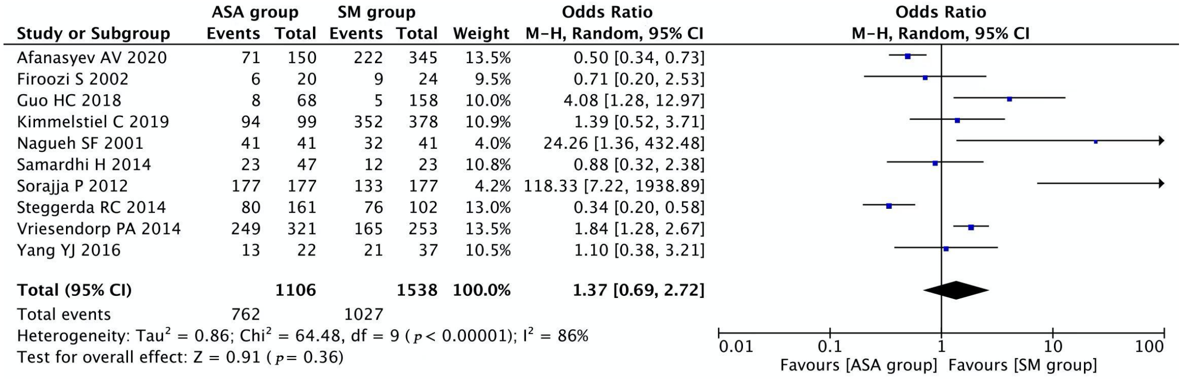

Figure S2. The comparisons of baseline interventricular septal diameter (IVSd, mm) and left ventricular end diastolic diameter (LVEDd, mm) between ASA groups and SM groups.

(a) comparison of baseline IVSd.

	ASA			SM				Mean Difference
**Study or Subgroup**	**Mean**	**SD**	**Total**	**Mean**	**SD**	**Total**	**Weight**	**IV, Fixed, 95% CI**
Cavigli L 2018	26	6	55	24	5	71	11.4%	2.00 [0.03, 3.97]
Firoozi S 2002	20	3	20	20	6	24	5.9%	0.00 [−2.74, 2.74]
Guo HC 2018	21.27	4.43	68	21.83	5.03	158	25.6%	−0.56 [−1.87, 0.75]
Qin JX 2001	23	4	25	24	6	26	5.7%	−1.00 [−3.79, 1.79]
Ralph−Edwards A 2005	22	5	50	25	6	46	9.0%	−3.00 [−5.22, −0.78]
Samardhi H 2014	20.5	5.6	47	22.9	10.4	27	2.5%	−2.40 [−6.64, 1.84]
ten Cate FJ 2010	23	4	91	24	5	40	14.3%	−1.00 [−2.75, 0.75]
Valeti US 2007	23	4.5	24	22	5	24	6.1%	1.00 [−1.69, 3.69]
van der Lee C 2005	21	4	43	23	4	29	12.4%	−2.00 [−3.88, −0.12]
Yang YJ 2016	25.1	4.9	22	25.8	4.4	37	7.1%	−0.70 [−3.19, 1.79]
**Total (95% CI)**			**445**			**482**	**100.0%**	**−0.68 [−1.35, −0.02]**

Heterogeneity: $Chi^2 = 15.80$, df = 9 ($p = 0.07$); $I^2 = 43\%$
Test for overall effect: Z = 2.01 ($p = 0.04$)

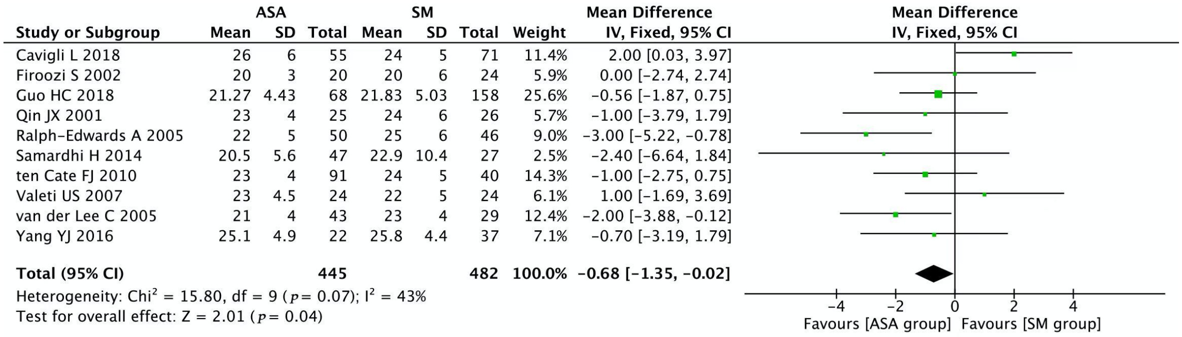

(b) comparison of baseline LVEDd.

	ASA			SM				Mean Difference
**Study or Subgroup**	**Mean**	**SD**	**Total**	**Mean**	**SD**	**Total**	**Weight**	**IV, Fixed, 95% CI**
Firoozi S 2002	42	4	20	40	7	24	6.4%	2.00 [−1.30, 5.30]
Qin JX 2001	40	8	25	44	7	26	4.1%	-4.00 [−8.13, 0.13]
Ralph−Edwards A 2005	43	5	48	43	6	44	13.5%	0.00 [−2.27, 2.27]
Sorajja P 2012	45	6	177	45	6	177	44.5%	0.00 [−1.25, 1.25]
ten Cate FJ 2010	40	4	91	39	4	40	31.5%	1.00 [−0.49, 2.49]
**Total (95% CI)**			**361**			**311**	**100.0%**	**0.28 [−0.56, 1.11]**

Heterogeneity: $Chi^2 = 6.31$, df = 4 ($p = 0.18$); $I^2 = 37\%$
Test for overall effect: Z = 0.66 ($p = 0.51$)

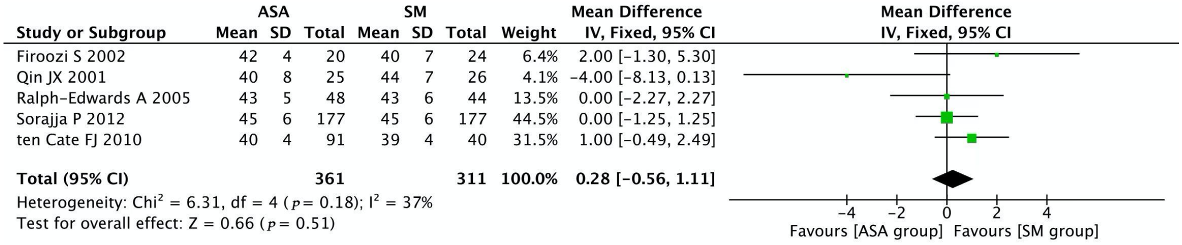

Figure S3. Evaluation of publication bias in the studies comparing ventricular arrythmias by funnelplot.

RR, risk ratio.

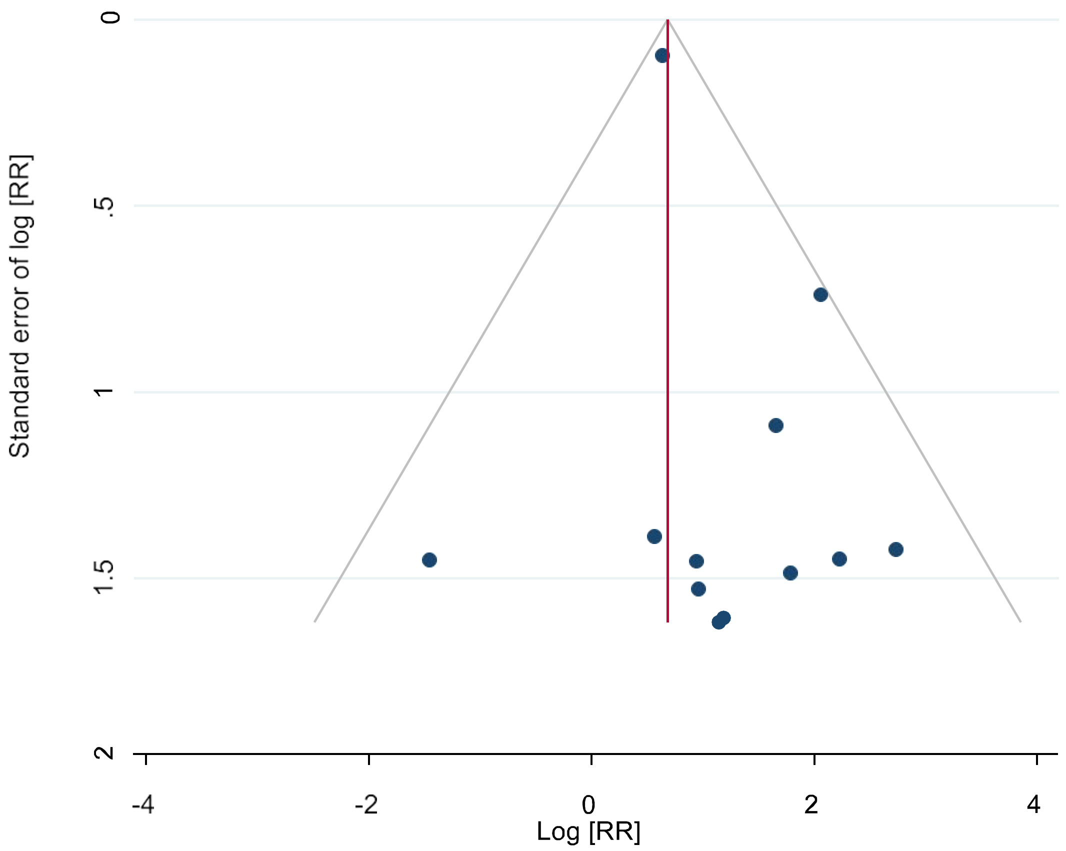

Figure S4. Evaluation of publication bias in the studies comparing sudden cardiac death (SCD)/resuscitated sudden cardiac arrest (SCA). RR, risk ratio.

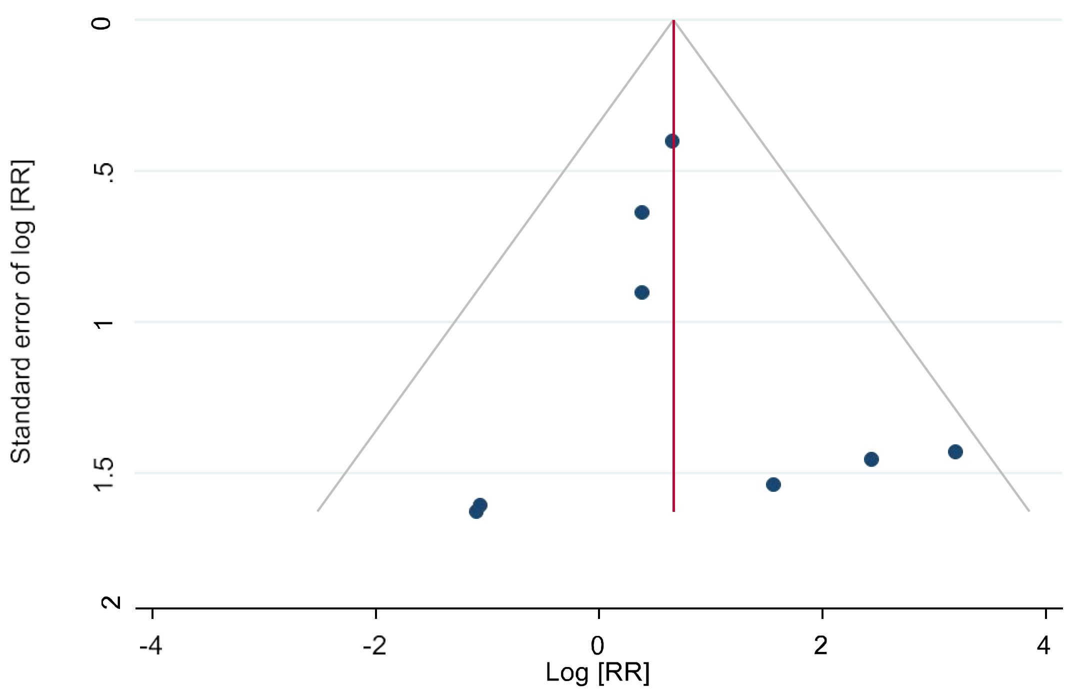

Figure S5. Meta-regression for interaction of LVOT pressure gradient reduction with VT/VF incidence between ASA and SM groups.

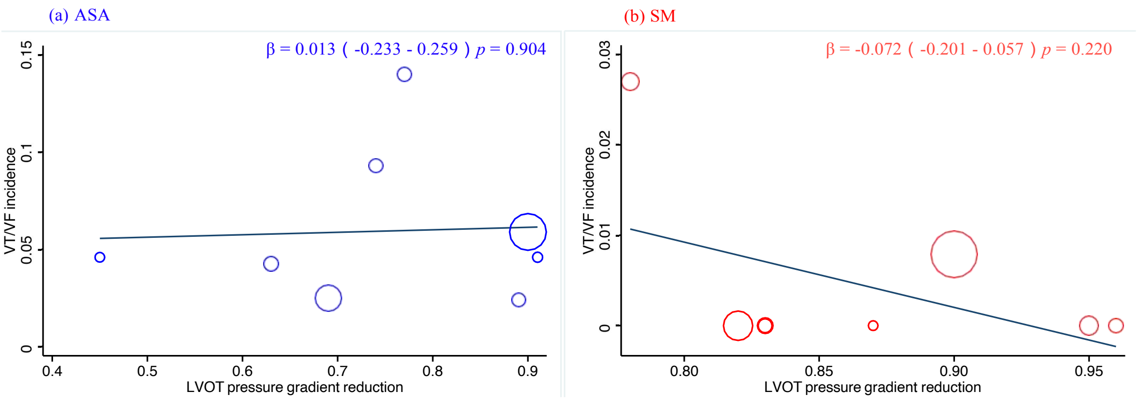

Figure S6. Meta-regression for interaction of baseline EF (%) with VT/VF incidence between ASA and SM groups.

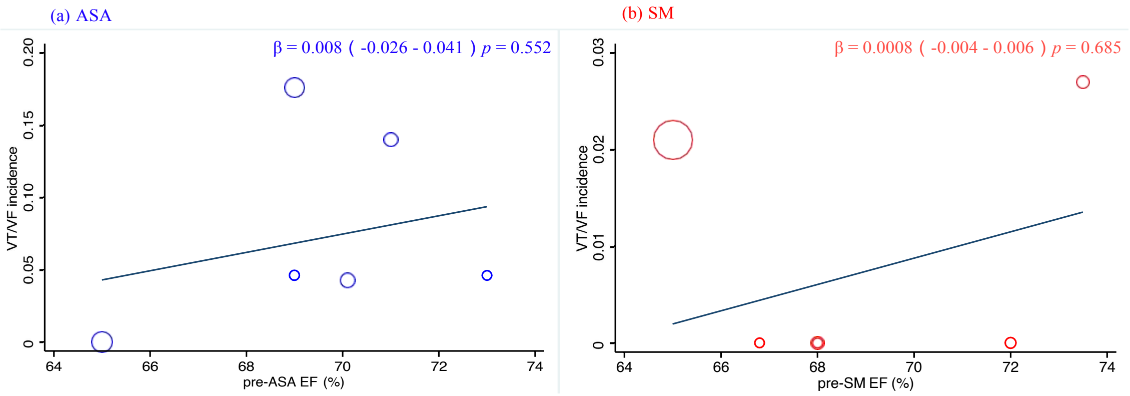

Figure S7. Meta-regression for interaction of baseline IVSd (mm) with VT/VF incidence between ASA and SM groups.

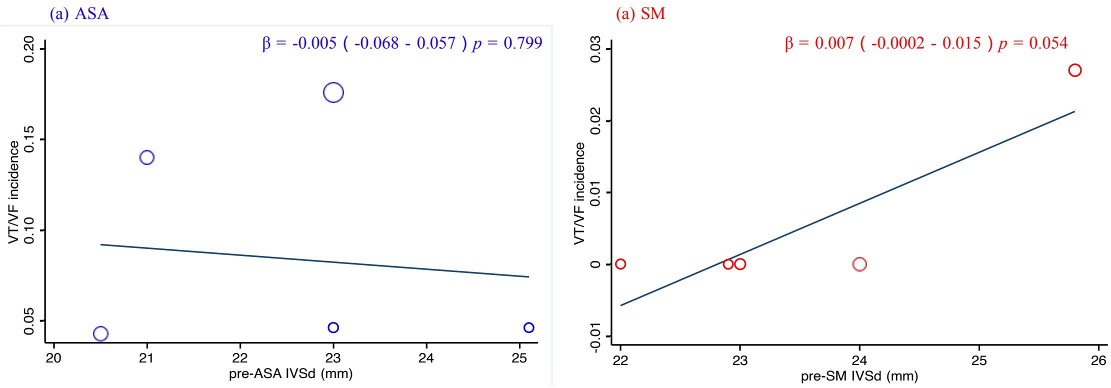

Figure S8. Meta-regression for interaction of baseline NYHA class III/IV proportion (%) with VT/VF incidence between ASA and SM groups.

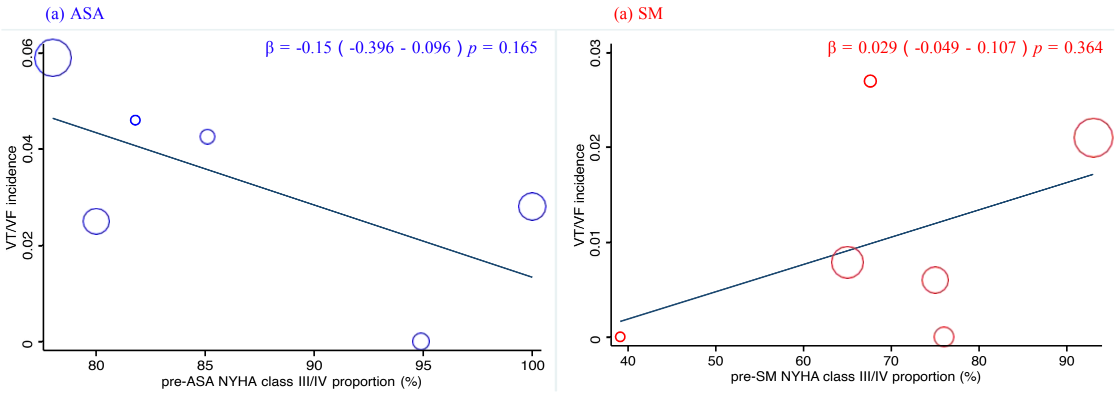

Supplement: Supplementary file 1 [file 2153-8174-23-12-391-s1.zip › 2153-8174-23-12-391-s1/SupplementaryFile.pdf]
